# [^18^F]FAPI-74 PET for Preoperative Assessment of Peritoneal Dissemination in Ovarian Cancer: A Case Series with Surgical and Histopathological Correlation

**DOI:** 10.3390/curroncol33070389

**Published:** 2026-06-29

**Authors:** Aasa Shimizu, Tadashi Watabe, Frederik L. Giesel, Yuriko Mori, Keita Asano, Yusaku Shimizu, Sadahiro Naka, Takashi Kamiya, Daisuke Katayama, Shinichiro Watanabe, Hiroki Kato, Kayako Isohashi, Mitsuaki Tatsumi, Noriyuki Tomiyama, Yasuto Kinose, Tadashi Iwamiya, Shinya Matsuzaki, Kenjiro Sawada, Michiko Kodama

**Affiliations:** 1 Department of Obstetrics and Gynecology, The University of Osaka Graduate School of Medicine, 2-2, Yamadaoka, Suita City 565-0871, Osaka, Japan; aasashimizu@gyne.med.osaka-u.ac.jp (A.S.); k.asano@gyne.med.osaka-u.ac.jp (K.A.); dgor0902@gyne.med.osaka-u.ac.jp (Y.S.); kinose0205@gyne.med.osaka-u.ac.jp (Y.K.); tiwamiya7@gyne.med.osaka-u.ac.jp (T.I.); zacky@gyne.med.osaka-u.ac.jp (S.M.); sawada.kenjiro.uw@ehime-u.ac.jp (K.S.); mkodama.med@osaka-u.ac.jp (M.K.); 2 Laboratory for Theranostics, Division of Clinical Translation, Institute for Radiation Sciences, The University of Osaka, 1-10, Yamadaoka, Suita City 565-0871, Osaka, Japan; 3 Department of Nuclear Medicine, Medical Faculty and University Hospital Düsseldorf, Heinrich Heine University Düsseldorf, Moorenstraße 5, 40225 Düsseldorf, Germany; frederik.giesel@med.uni-duesseldorf.de (F.L.G.); yuriko.mori@med.uni-duesseldorf.de (Y.M.); 4 Department of Pharmacy, The University of Osaka, 2-15, Yamadaoka, Suita City 565-0871, Osaka, Japan; naka-s@office.osaka-u.ac.jp; 5 Department of Medical Technology, The University of Osaka, 2-2, Yamadaoka, Suita City 565-0871, Osaka, Japan; kamiya-t@office.osaka-u.ac.jp; 6 Department of Radiology, The University of Osaka Graduate School of Medicine, 2-2, Yamadaoka, Suita City 565-0871, Osaka, Japan; katayama.daisuke.med@osaka-u.ac.jp (D.K.); s-watanabe@radiol.med.osaka-u.ac.jp (S.W.); kato.hiroki.med@osaka-u.ac.jp (H.K.); isohashi.kayako.med@osaka-u.ac.jp (K.I.); tomiyama@radiol.med.osaka-u.ac.jp (N.T.); 7 Department of Diagnostic Radiology, Juntendo University Graduate School of Medicine, 2-1-1, Hongo, Bunkyo-ku, Tokyo 113-0033, Japan; m.tatsumi.pz@juntendo.ac.jp; 8 Department of Obstetrics and Gynecology, Sakai City Medical Center, 1-1-1, Ebaraji-cho, Nishi-ku, Sakai City 593-8304, Osaka, Japan; 9 Department of Obstetrics and Gynecology, Ehime University Graduate School of Medicine, Shitsukawa, Toon 791-0204, Ehime, Japan

**Keywords:** FAPI-PET, FAP, ovarian cancer, cancer-associated fibroblasts, MIS

## Abstract

Background/Objectives: Accurate preoperative assessment of peritoneal dissemination is essential in ovarian cancer because it influences surgical strategy and the achievement of complete gross resection. However, [^18^F]FDG-PET may be limited in detecting lesions with low glycolytic activity and in differentiating malignancy from inflammatory changes. This case series evaluated the clinical relevance of [^18^F]FAPI-74 PET/CT for preoperative assessment of peritoneal dissemination in ovarian cancer. Methods: Four patients underwent [^18^F]FAPI-74 PET/CT as part of preoperative evaluation, with comparison to [^18^F]FDG-PET/CT when available. Imaging findings were correlated with intraoperative observations and histopathological results, including immunohistochemical assessment of fibroblast activation protein and α-smooth muscle actin. Results: FAPI-PET detected peritoneal dissemination not identified by FDG-PET in several cases, including occult metastasis confirmed histologically and additional lesions after neoadjuvant chemotherapy. FAPI-avid lesions showed stromal activation on immunohistochemistry, supporting the biological basis of FAPI uptake. In one case, additional FAPI uptake may have been partly influenced by inflammatory changes associated with bloody ascites. Conclusions: FAPI-PET may provide complementary information by visualizing stromal components of ovarian cancer and may support preoperative mapping of peritoneal dissemination, although interpretation should consider inflammatory conditions.

## Simple Summary

Ovarian cancer is the leading cause of death among gynecologic malignancies, and complete tumor resection is essential for successful treatment. To accomplish this surgery, accurate evaluation of tumor spread before surgery is important. FDG-PET is commonly used for preoperative imaging, but some peritoneal lesions are difficult to detect. FAPI-PET is a newer imaging method that visualizes activated fibroblasts within the tumor microenvironment. We describe four ovarian cancer patients who underwent FAPI-PET and FDG-PET before surgery. These findings suggest that FAPI-PET may improve preoperative assessment of peritoneal dissemination in ovarian cancer.

## 1. Introduction

Positron emission tomography (PET) using 18F-fluorodeoxyglucose ([^18^F]FDG) is a widely used radiotracer in gynecologic oncology imaging for disease staging, assessment of treatment response, and detection of recurrence [1]. FDG-PET provides valuable metabolic information, particularly in advanced-stage disease; however, its diagnostic performance is limited in lesions with low glycolytic activity, as some cancers exhibit metabolic inactivity or rely on alternative energy sources for survival. In addition, FDG uptake does not reliably distinguish malignant lesions from inflammation or fibrosis, both of which are frequently encountered in the peritoneal cavity [2,3]. FDG-based measurements are also susceptible to variability related to patient preparation, including fasting and resting before imaging [4]. Ovarian cancer remains one of the leading causes of cancer-related death among women worldwide and is the most lethal gynecologic malignancy. Owing to nonspecific symptoms and the lack of effective screening strategies, most patients are diagnosed with advanced-stage disease characterized by extensive peritoneal dissemination, which remains a major determinant of prognosis and treatment planning [5]. However, accurate preoperative assessment of peritoneal dissemination remains a critical challenge in ovarian cancer because it directly influences surgical strategy and the likelihood of achieving complete gross resection (R0) [6].

In parallel, the indications for minimally invasive surgery (MIS) in both primary debulking surgery (PDS) and interval debulking surgery (IDS) have gradually expanded in recent years [7,8,9]. Contemporary retrospective data suggest that, in carefully selected patients, particularly those demonstrating a favorable response to neoadjuvant chemotherapy, MIS for IDS may achieve survival outcomes comparable to those of laparotomy while maintaining acceptable cytoreduction rates, and prospective randomized evaluation is currently underway [8,10]. As MIS becomes more widely adopted, rigorous preoperative mapping of peritoneal disease and nodal involvement has become increasingly important to ensure that complete macroscopic resection can be performed safely and consistently through a minimally invasive approach.

The tumor microenvironment of ovarian cancer is characterized by abundant stromal components, among which cancer-associated fibroblasts (CAFs) play a central role [11,12,13]. CAFs actively promote tumor invasion, peritoneal dissemination, extracellular matrix remodeling, and immunosuppression through paracrine signaling pathways, including transforming growth factor-β. Fibroblast activation protein (FAP), a serine protease selectively expressed on activated fibroblasts, is highly upregulated in CAFs while showing minimal expression in normal adult tissues, making it an attractive target for molecular imaging of the tumor stroma [14].

Radiolabeled fibroblast activation protein inhibitors (FAPI) have enabled PET imaging of stromal activation rather than glucose metabolism. FAPI-PET has demonstrated rapid clearance from normal tissues, low background uptake, and high tumor-to-background ratios across a variety of malignancies [15,16]. These characteristics suggest that FAPI-PET may be particularly advantageous in ovarian cancer, in which diffuse peritoneal dissemination and stromal-rich tumor architecture often limit the sensitivity of FDG-PET and contrast-enhanced computed tomography (CT).

Since 2020, several studies have explored the application of FAPI-PET in gynecologic malignancies, including ovarian cancer [17,18]. These reports suggest improved visualization of peritoneal lesions and metastatic sites compared with FDG-PET. However, the currently available data remain preliminary, and direct correlations among FAPI uptake, intraoperative findings, and histopathological validation have been limited.

In this study, we present a case series of patients with ovarian cancer who underwent FAPI-PET/CT as part of the preoperative evaluation, with direct comparison to FDG-PET/CT when available. Imaging findings were systematically correlated with intraoperative observations and histopathological analyses, including immunohistochemical assessment of FAP expression. Through this multimodal approach, we aimed to clarify the clinical relevance of FAPI-PET/CT for evaluating peritoneal dissemination and supporting surgical decision-making in ovarian cancer.

## 2. Case Presentations

Across the four cases, FAPI-PET appeared to provide additional diagnostic information regarding peritoneal dissemination compared with FDG-PET, particularly for lesions not identified on conventional imaging or during intraoperative inspection. In Case 1, FAPI-PET detected occult peritoneal metastasis that was subsequently confirmed histopathologically. In Case 2, FAPI-PET identified additional peritoneal lesions following neoadjuvant chemotherapy, corresponding to CAF-rich tumor microenvironments. In Case 3, although FDG-PET demonstrated higher uptake, this difference was attributable to the timing of imaging, whereas FAPI-PET more accurately reflected the actual distribution of disease. In contrast, Case 4 highlighted a potential limitation of FAPI-PET, in which reactive uptake may have reflected inflammatory changes associated with bloody ascites. Collectively, these findings suggest that FAPI-PET provides valuable information for preoperative mapping of peritoneal disease, while still requiring careful interpretation in specific biological contexts. The clinical characteristics of all patients are summarized in Table 1. Quantitative PET parameters, including SUVmax values for representative lesions on both FDG-PET and FAPI-PET, are summarized in Table 2. Details of the PET/CT acquisition protocols are provided in the Supplementary Materials, and the administered activities of both tracers for each patient are summarized in Supplementary Table S1.

### 2.1. Case 1

**Figure 1:**
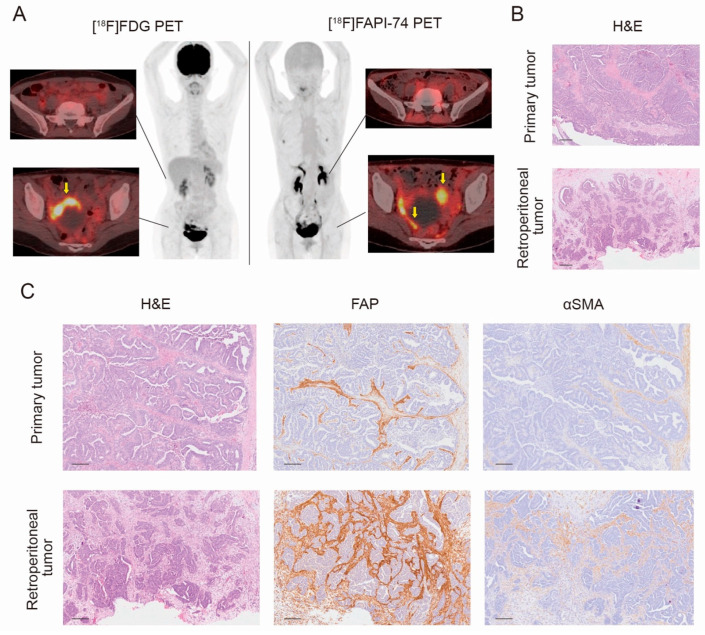
Multimodal imaging and histopathological evaluation in Case 1: Detection of occult peritoneal metastasis by FAPI-PET. (**A**) Comparison of maximum-intensity projection images and axial PET/CT images. The (**left**) panels show [^18^F]FDG-PET, and the (**right**) panels show [^18^F]FAPI-74-PET. Yellow arrows indicate tumor lesions. (**B**) Hematoxylin and eosin (H&E) staining of the resected tumor from the primary tumor and peritoneal metastases. (**C**) Evaluation of cancer-associated fibroblast infiltration using H&E and immunohistochemical staining for fibroblast activation protein and α-smooth muscle actin. Scale bars represent 200 μm. H&E, hematoxylin and eosin. FAP, fibroblast activation protein. αSMA, α-smooth muscle actin.

A 51-year-old woman with ovarian cancer (clinical FIGO stage IB) was referred for preoperative imaging evaluation. Contrast-enhanced CT revealed a localized ovarian tumor without evidence of peritoneal dissemination or distant metastasis. FDG-PET demonstrated mild tracer uptake confined to the primary ovarian lesion, with no abnormal uptake in the peritoneum or distant sites. In contrast, FAPI-PET suggested possible peritoneal dissemination in the Douglas pouch (Figure 1A). The patient subsequently underwent primary debulking surgery. Although no obvious macroscopic dissemination was identified intraoperatively, invasion of the rectal serosa was suspected, and low anterior resection was additionally performed. Histopathological examination confirmed high-grade serous ovarian carcinoma (HGSOC) and revealed metastatic cancer cells in the peritoneum of the Douglas pouch, corresponding to the lesion suspected only on FAPI-PET (Figure 1B). Immunohistochemical (IHC) analysis further demonstrated stronger expression of FAP and αSMA in the retroperitoneal tumor than in the primary tumor, suggesting a higher abundance of CAFs at the metastatic site (Figure 1C).

### 2.2. Case 2

**Figure 2:**
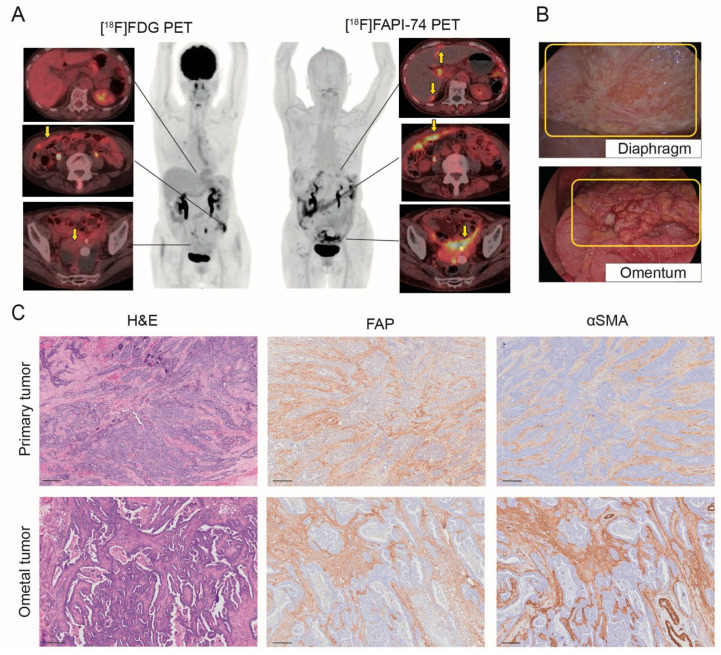
Multimodal imaging and histopathological evaluation in Case 2: Residual peritoneal disease detection after neoadjuvant chemotherapy. (**A**) Comparison of maximum-intensity projection images and axial PET/CT images. The (**left**) panels show [18F]FDG-PET, and the (**right**) panels show [18F]FAPI-74-PET. Yellow arrows indicate tumor lesions. (**B**) Intraoperative images confirming disseminated lesions corresponding to areas of increased FAPI uptake in the diaphragm and omentum. Yellow squares indicate the dissemination sites. (**C**) Representative histological findings of the primary and metastatic lesions. H&E and immunohistochemical staining for fibroblast activation protein and α-smooth muscle actin. Scale bars represent 200 μm. H&E, hematoxylin and eosin. FAP, fibroblast activation protein. αSMA, α-smooth muscle actin.

A 79-year-old woman with advanced ovarian cancer (clinical FIGO stage IIIC) received four cycles of neoadjuvant chemotherapy with paclitaxel and carboplatin, followed by planned interval debulking surgery. FDG-PET and FAPI-PET were performed preoperatively to evaluate residual disease. FDG-PET demonstrated moderate uptake in residual tumor lesions, including the primary tumor and omental lesions in the subhepatic and perisplenic regions, but showed no abnormal uptake in the diaphragm or parietal peritoneum. In contrast, FAPI-PET revealed intense uptake not only in the residual primary tumor but also at additional peritoneal sites, including the diaphragm and parietal peritoneum, which were not detected by FDG-PET (Figure 2A). Subsequent interval debulking surgery confirmed disseminated lesions in these regions, closely corresponding to the sites of increased FAPI uptake (Figure 2B), with optimal cytoreduction achieved. Histopathological examination of the resected specimens confirmed HGSOC. Hematoxylin and eosin (H&E) staining and IHC for FAP and αSMA indicated that the resected tumor retained abundant CAFs within the tumor microenvironment, despite prior neoadjuvant chemotherapy, consistent with the observed FAPI uptake (Figure 2C).

### 2.3. Case 3

**Figure 3:**
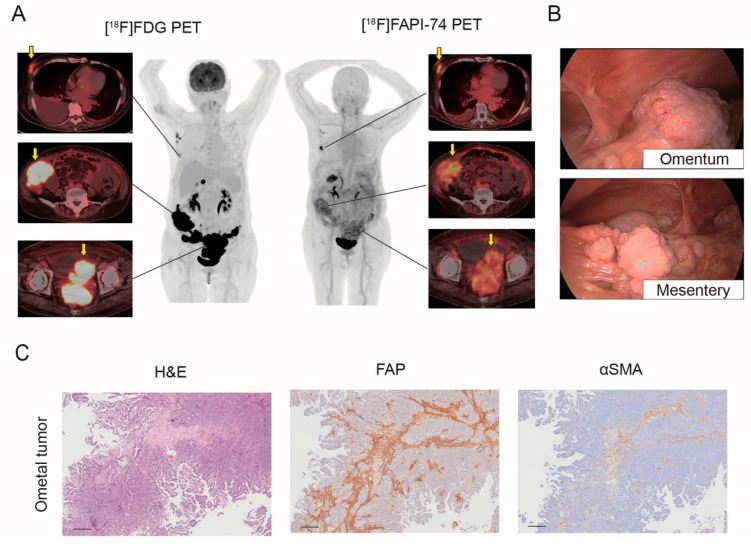
Multimodal imaging and histopathological evaluation in Case 3: Higher FDG uptake compared with FAPI-PET. (**A**) Comparison of maximum-intensity projection images and axial PET/CT images. The (**left**) panels show [18F]FDG-PET, and the (**right**) panels show [18F]FAPI-74-PET. Yellow arrows indicate tumor lesions. (**B**) Intraoperative images confirming disseminated lesions corresponding to areas of increased FAPI uptake in the omentum and mesentery. (**C**) Representative histological findings of the peritoneal dissemination. H&E and immunohistochemical staining for fibroblast activation protein. Scale bars represent 200 μm. H&E, hematoxylin and eosin. FAP, fibroblast activation protein. αSMA, α-smooth muscle actin.

A 74-year-old woman with ovarian cancer (clinical FIGO stage IIIC) underwent preoperative imaging evaluation with contrast-enhanced CT and FAPI-PET. Contrast-enhanced CT showed no definitive evidence of mesenteric tumor involvement, although subtle abnormalities raised suspicion for minimal peritoneal dissemination. In contrast, FAPI-PET demonstrated marked tracer uptake along the mesentery and omentum, suggesting extensive mesenteric dissemination (Figure 3A). The patient subsequently underwent staging laparoscopy to evaluate tumor spread and confirm histology, during which peritoneal dissemination involving both the mesentery and omentum was clearly identified (Figure 3B). The macroscopic distribution of disease closely matched the areas of increased FAPI uptake observed preoperatively. Histopathological examination of resected specimens confirmed HGSOC with peritoneal dissemination. Although the distribution of disease corresponded closely to the areas of increased FAPI uptake, treatment planning was ultimately guided by laparoscopic assessment and conventional imaging findings. Following diagnostic laparoscopy and before initiation of systemic therapy, FDG-PET was performed and showed higher tracer uptake in the tumor lesions compared with that of the prior FAPI-PET, suggesting that these tumors had high proliferative activity and aggressive malignant potential. In addition, IHC analysis of the resected disseminated lesions demonstrated strong expression of FAP and positive staining for αSMA, consistent with a CAF-rich tumor microenvironment (Figure 3C). Based on the laparoscopic findings, primary debulking surgery was deferred, and the patient subsequently received three cycles of neoadjuvant chemotherapy with paclitaxel, carboplatin, and bevacizumab.

### 2.4. Case 4

**Figure 4:**
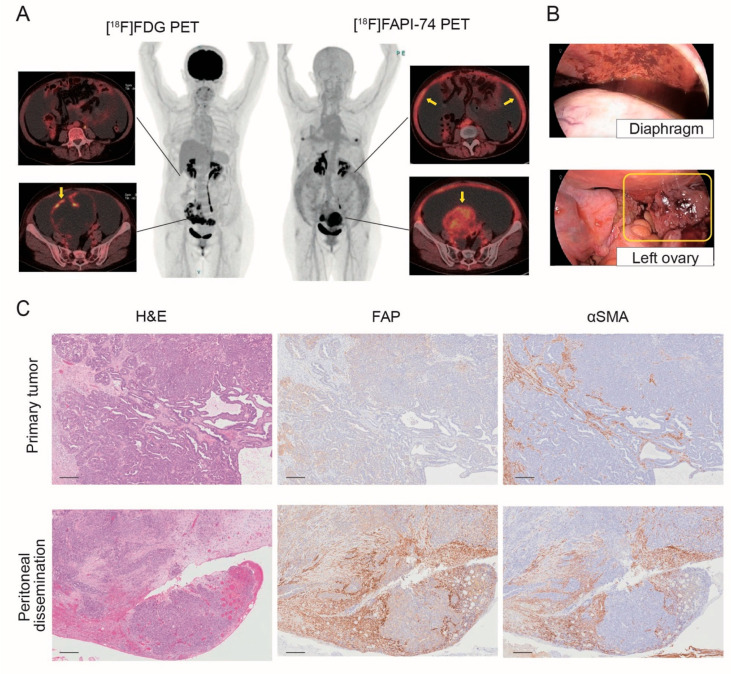
Multimodal imaging and histopathological evaluation in Case 4: FAPI-PET uptake in bloody ascites. (**A**) Comparison of maximum-intensity projection images and axial PET/CT images. The (**left**) panels show [18F]FDG-PET, and the (**right**) panels show [18F]FAPI-74-PET. Yellow arrows indicate tumor lesions. (**B**) Intraoperative images confirming disseminated lesions corresponding to areas of increased FAPI uptake in the diaphragm and omentum. Yellow squares indicate the dissemination sites. (**C**) Representative histological findings of the primary and metastatic lesions. H&E and immunohistochemical staining for fibroblast activation protein and α-smooth muscle actin. Scale bars represent 200 μm. H&E, hematoxylin and eosin. FAP, fibroblast activation protein. αSMA, α-smooth muscle actin.

A 64-year-old woman with suspected advanced ovarian cancer (clinical FIGO stage IIIA) underwent both FDG-PET and FAPI-PET as part of the preoperative evaluation. FDG-PET demonstrated uptake limited to peritoneal lesions in the Douglas pouch. In contrast, FAPI-PET revealed not only intense uptake in the Douglas pouch but also mild diffuse uptake along the parietal peritoneum (Figure 4A). The patient initially underwent staging laparoscopy, during which bloody ascites was observed. No dissemination was identified along the parietal peritoneum on laparoscopic evaluation, and the overall intra-abdominal disease burden appeared limited and resectable, prompting conversion to primary debulking surgery (Figure 4B). At laparotomy, comprehensive inspection of the abdominal cavity confirmed peritoneal dissemination in the Douglas pouch but not along the parietal peritoneum. These findings indicated that the overall tumor burden was lower than initially suggested by FAPI-PET, and complete cytoreduction was achieved. Histopathological examination confirmed HGSOC with abundant stromal components in the resected peritoneal lesions. IHC analysis demonstrated strong expression of FAP and positive staining for αSMA in the tumor stroma of the FAPI-avid lesions, consistent with a CAF-rich tumor microenvironment (Figure 4C). Following surgery, the patient received adjuvant chemotherapy with paclitaxel, carboplatin, and bevacizumab.

## 3. Discussion

Accurate preoperative assessment of peritoneal dissemination remains a central challenge in managing ovarian cancer, particularly as indications for MIS continue to expand. Because surgical planning increasingly depends on precise delineation of disease extent to achieve complete macroscopic resection, imaging modalities capable of identifying occult peritoneal lesions have become increasingly important in clinical practice [8,9,19]. In this context, our case series suggests that FAPI-PET may improve detection of peritoneal dissemination through stromal-targeted imaging, while also underscoring limitations that should be considered in clinical decision-making. Consistent with previous reports, FAPI-PET showed higher sensitivity than FDG-PET for detecting peritoneal lesions in several cancer types [20].

In Case 1, FAPI-PET identified occult peritoneal metastasis that was not detected by contrast-enhanced CT or FDG-PET but was subsequently confirmed histologically. This finding underscores the ability of FAPI-PET to detect stromal activation associated with early metastatic niches, which may not yet exhibit sufficient glycolytic activity to be visualized by FDG-PET [21,22]. Similarly, in Case 2, FAPI-PET identified additional peritoneal lesions after neoadjuvant chemotherapy, suggesting that CAF-rich tumor microenvironments may persist despite cytotoxic treatment and remain detectable by FAP-based imaging.

A key strength of the present study is the integration of imaging findings with histopathological validation, including immunohistochemical assessment of FAP and αSMA expression. Across cases, FAPI-avid lesions demonstrated strong expression of FAP and αSMA, supporting the biological basis of FAPI uptake as a marker of CAF activity. Notably, FAP expression was higher in metastatic and disseminated lesions than in the primary tumor, corresponding to the increased FAPI uptake observed at these sites and further supporting the role of FAPI-PET as an imaging modality that reflects a CAF-rich tumor microenvironment. CAFs are known to contribute to ovarian cancer progression, promoting extracellular matrix remodeling, suppression of immune cell infiltration, tumor invasion, and peritoneal dissemination [14,23,24]. Our findings provide direct clinical evidence linking stromal activation to FAPI-PET signal in ovarian cancer and extend previous imaging-based observations through spatial and pathological validation.

However, our results also highlight important limitations of FAPI-PET. In Case 4, FAPI-PET showed widespread mild uptake along the parietal peritoneum, whereas laparoscopic and intraoperative findings demonstrated limited disease primarily confined to the Douglas pouch. This discrepancy suggests that FAPI-PET may yield false-positive findings under certain conditions. One possible explanation is the presence of bloody ascites, which may induce inflammatory activation of fibroblasts along the peritoneal surfaces. Activated fibroblasts in inflammatory or reparative processes can also express FAP, potentially leading to non-specific tracer accumulation. This observation indicates that FAPI-PET uptake does not exclusively reflect malignant involvement and may be influenced by the underlying inflammatory microenvironment. These findings highlight the importance of interpreting FAPI-PET results within the broader clinical context. In particular, diffuse peritoneal uptake in the presence of bloody ascites or suspected inflammatory conditions should be interpreted cautiously and correlated with conventional imaging and surgical findings whenever possible. Future studies are warranted to establish imaging criteria that improve the distinction between malignant and inflammation-related FAPI uptake.

Collectively, these findings suggest that FAPI-PET provides information complementary to that of FDG-PET by visualizing stromal components of the tumor microenvironment. This feature may be particularly advantageous for detecting peritoneal dissemination in ovarian cancer, in which tumor spread is often diffuse and associated with a prominent stromal component. Although FAPI-PET may contribute additional information regarding disease distribution, its clinical utility for guiding treatment selection and surgical strategy remains to be determined in larger prospective studies.

Nevertheless, the present study has several limitations. This is a small case series, and the findings require validation in larger prospective cohorts. Furthermore, quantitative analysis of tracer uptake and standardized imaging protocols were not systematically evaluated. FDG-PET and FAPI-PET were not always performed under identical clinical conditions; for example, in Case 3, the interval between the two imaging studies may have influenced lesion detectability, potentially introducing bias into the comparison between the modalities. Finally, all cases included in this study were histologically confirmed as HGSOC. Because stromal composition and FAP expression may differ among ovarian cancer histological subtypes, the findings observed in this study may not be directly applicable to other histological types. Validation of additional histological subtypes of ovarian cancer is required to determine the applicability of FAPI-PET across other histological subtypes of ovarian cancer.

At present, FAPI-PET may be particularly valuable in cases with equivocal findings on conventional imaging. Suspicious FAPI-positive lesions should be interpreted in conjunction with other imaging modalities and, when clinically indicated, confirmed by surgical assessment. Larger prospective studies incorporating quantitative PET metrics, spatial transcriptomics, and detailed stromal characterization are needed to define optimal patient selection, establish standardized clinical workflows, and further clarify the role of FAPI-PET in ovarian cancer.

## 4. Conclusions

FAPI-PET enables visualization of CAF-rich tumor microenvironments and may improve the detection of peritoneal dissemination in ovarian cancer. However, its interpretation requires consideration of biological context, including inflammatory changes and imaging timing. FAPI-PET represents a promising tool for preoperative disease mapping and surgical planning, particularly in the evolving landscape of minimally invasive ovarian cancer surgery.

## Figures and Tables

**Table 1:** Patient characteristics: PDS, primary debulking surgery; IDS, interval debulking surgery; HGSOC, high-grade serous ovarian carcinoma; LN, lymph node; FDG, fluorodeoxyglucose; FAPI, fibroblast activation protein inhibitor.

Case	Age (Years)	Clinical Stage (FIGO)	Treatment Setting	Final Pathology	Surgical Achievement	FDG-PET Findings	Additional FAPI-PET Findings
1	51	IIIC	PDS	HGSOC	R0	Bilateral ovarian lesions	Peritoneal dissemination
2	79	IIIC	IDS	HGSOC	Optimal	Peritoneal dissemination, spleen	Diaphragmatic dissemination
3	74	IVB	Staging laparoscopy	HGSOC	-	Peritoneal dissemination, right breast lesion, right axillary LN	Similar to FDG findings
4	63	IIIC	PDS	HGSOC	R0	Bilateral ovarian cancer, peritoneal dissemination	Pelvic wall peritoneal dissemination

**Table 2:** SUVmax for representative lesions in each case. FDG, fluorodeoxyglucose. FAPI, fibroblast activation protein inhibitor. PET, positron emission tomography.

	Tumor Lesion	FDG-PET	FAPI-PET
Case 1	Primary tumor (Right)	12.55	11.82
	Primary tumor (Left)	6.36	4.75
	Peritoneal dissemination	5.35	9.7
Case 2	Primary tumor	1.72	8.55
	Omental tumor	2.56	8.84
	Peritoneal dissemination	3.43	7.34
	Diaphragm tumor	3.05	5.81
Case 3	Primary tumor	21.84	7.51
	Omental tumor	24.56	5.68
	Peritoneal dissemination	23.46	6.22
	Right breast	4.24	7.34
	Right axillary lymph node	6.22	4.94
Case 4	Primary tumor	25.25	13.54
	Parietal peritoneum	1.4	3.96

## Data Availability

The data underlying this article will be shared upon reasonable request to the corresponding author.

## Supplementary Materials

The following supporting information can be downloaded at: https://www.mdpi.com/article/10.3390/curroncol33070389/s1. Table S1: Administered activities of FDG and FAP tracers for each PET examination. FDG, fluorodeoxyglucose. FAPI, fibroblast activation protein inhibitor. PET, Positron emission tomography.

## Abbreviations

The following abbreviations are used in this manuscript:

CAF	Cancer-associated fibroblast
CT	Computed tomography
FAP	Fibroblast activation protein
FAPI	Fibroblast activation protein inhibitor
FDG	Fluorodeoxyglucose
FIGO	International Federation of Gynecology and Obstetrics
HGSOC	High-grade serous ovarian carcinoma
IDS	Interval debulking surgery
IHC	Immunohistochemistry
MIS	Minimally invasive surgery
PET	Positron emission tomography
PDS	Primary debulking surgery
αSMA	α-smooth muscle actin
