# Supplementary material for: [18F]FAPI-74 PET for Preoperative Assessment of Peritoneal Dissemination in Ovarian Cancer: A Case Series with Surgical and Histopathological Correlation
Source: Curr Oncol. 2026 Jun 29;33(7):389. doi: 10.3390/curroncol33070389 (PMC13409096; doi:10.3390/curroncol33070389)
Supplement: Supplementary file 1 [file curroncol-33-00389-s001.zip › curroncol-4349380-supplementary.pdf]

SUPPLEMENTARY MATERIALS

Materials and methods

Study design and patients

This retrospective case series analyzed ovarian cancer cases included in the clinical trial registered at ClinicalTrials.gov (Evaluation Using FAPI-PET Targeting Cancer-associated Fibroblasts, NCT05442151). Patients underwent [^{18}F]FAPI-74 PET/CT as part of the registered clinical study, and clinical, surgical, imaging, and histopathological findings were retrospectively reviewed. All patients provided written informed consent for undergoing FAPI-PET/CT and for the retrospective use of their clinical data for research purposes. This study was conducted in accordance with the Declaration of Helsinki and approved by the Institutional Review Board of The University of Osaka (no. 21472).

Imaging procedures

All patients underwent FAPI-PET/CT, and FDG-PET/CT was performed when clinically indicated. For FDG-PET/CT, patients fasted for at least 4 before tracer injection, and imaging was performed approximately 60 min after administration of [^{18}F]FDG. [^{18}F]FAPI-74 solution was synthesized using CFN-MPS200 (Sumitomo Heavy Industries) according to our previous report [1]. FAPI-PET/CT was performed 60 min after intravenous injection of [^{18}F]FAPI-74 using a Biograph Vision 600 scanner (Siemens Healthineers, Erlangen, Germany) in accordance with the institutional protocol. The administered activities of [^{18}F]FDG and [^{18}F]FAPI 74 for each examination are summarized in Supplementary Table S1. PET/CT images were reviewed by board-certified nuclear medicine physicians. Tracer uptake distribution and intensity were evaluated qualitatively, with particular attention to peritoneal dissemination, omental involvement, and nodal disease. Image interpretation was performed by consensus among nuclear medicine physicians, who were blinded to the surgical and histopathological findings at the time of evaluation.

Surgical evaluation

All patients underwent surgical evaluation, including staging laparoscopy and/or cytoreductive surgery. The extent and distribution of peritoneal dissemination were recorded intraoperatively and compared with preoperative imaging findings. The intraoperative distribution of disease was systematically assessed and recorded by anatomical site. Surgical outcomes, including the

completeness of cytoreduction, were also documented. Tumor stage was determined according to the International Federation of Gynecology and Obstetrics (FIGO) 2014 staging system.

Histopathological and immunohistochemical analysis

Resected tumor specimens were fixed in formalin and embedded in paraffin. Hematoxylin and eosin (H&E) staining was performed for routine histopathological evaluation, and staining intensity and distribution were assessed. Immunohistochemical staining was performed using antibodies against FAP and α-smooth muscle actin (αSMA) to assess the presence of CAFs within the tumor microenvironment. A total of four cases were included in this study. Four-µm-thick sections were cut from the formalin-fixed tissue and mounted on silane-coated glass slides. The slides were incubated with primary antibodies against FAP (Abcam; ab207178) at 1:150 and αSMA (Dako; M0851) at 1:200 for 1 h at 20℃–25℃. After washing with phosphate-buffered saline containing 0.01% Tween-20, the sections were stained using LiquidDABþSubstrate Chromogen System (Dako; K3467) and then counterstained with Carrazzi's hematoxylin. Staining patterns were evaluated qualitatively and correlated with imaging findings, particularly FAPI uptake. All histopathological diagnoses were reviewed and confirmed by an experienced gynecologic pathologist.

Data analysis

Imaging findings from FDG-PET and FAPI-PET were compared with intraoperative and histopathological results. Concordance between tracer uptake and actual tumor distribution was assessed descriptively across cases.

Supplementary Table

	FDG-PET (MBq)	FAPI-PET (MBq)
Case 1	163	200
Case 2	156	210
Case 3	235	225
Case 4	212	234

Supplementary Table S1. Administered activities of FDG and FAP tracers for each PET examination. *F*DG, fluorodeoxyglucose. FAPI, fibroblast activation protein inhibitor. *PET, P*ositron emission tomography.

Reference

1. Naka S, Watabe T, Lindner T, Cardinale J, Kurimoto K, Moore M, et al. One-pot and one-step automated radio-synthesis of [18F]AlF-FAPI-74 using a multi purpose synthesizer: a proof-of-concept experiment. EJNMMI Radiopharm Chem. 2021, 6, 28. https://doi.org/10.1186/s41181-021-00142-z
